# Prognostic significance of the aspartate aminotransferase to lymphocyte ratio index in patients with acute myocardial infarction

**DOI:** 10.1002/iid3.1306

**Published:** 2024-06-18

**Authors:** Huidi Liu, Fan Zhao, Jun Yin, Taimin Liu, Bo Liu

**Affiliations:** ^1^ Department of Cardiology, Wuhan Sixth Hospital Affiliated Hospital of Jianghan University Wuhan Hubei China

**Keywords:** acute myocardial infarction, alanine aspartate aminotransferase to lymphocyte ratio, MIMIC‐III database, prognosis

## Abstract

**Background:**

This study aimed to investigate the clinical value and prognostic significance of the alanine aspartate aminotransferase‐to‐lymphocyte ratio index (ALRI) in patients diagnosed with acute myocardial infarction (AMI).

**Methods:**

Clinical indices of patients with AMI were collected from the Medical Information Mark for Intensive Care (MIMIC) III database and Wuhan Sixth Hospital. Cox regression analysis was used to explore whether ALRI was a risk factor for a worse prognosis in patients with AMI, and a nomogram including ALRI was created to estimate its predictive performance for 28‐day mortality.

**Results:**

Based on clinical data from the MIMIC‐III database, we found that a high ALRI was closely associated with a variety of clinical parameters. It was an important risk factor for 28‐day survival in patients with AMI (HR = 5.816). ALRI had a high predictive power for worse 28‐day survival in patients with AMI (area under the curve [AUC] = 0.754). Additionally, we used clinical data from the Wuhan Sixth Hospital to verify the predictive power of ALRI in patients with AMI, and a high level of ALRI remained an independent risk factor for worse survival in patients with AMI (HR = 4.969). The AMI nomogram, including ALRI, displayed a good predictive performance for 28‐day mortality in both the MIMIC‐III (AUC = 0.826) and Wuhan Sixth Hospital cohorts (AUC = 0.795).

**Conclusion:**

The ALRI is closely related to the survival outcomes of patients with newly diagnosed AMI, indicating that it could serve as a novel biomarker for risk stratification such patients.

## Graphical abstract

We aimed to explore the correlation between aminotransferase‐to‐lymphocyte ratio index (ALRI) and clinical features of acute myocardial infarction patients, and investigate the prognostic significance of ALRI using two data sets.

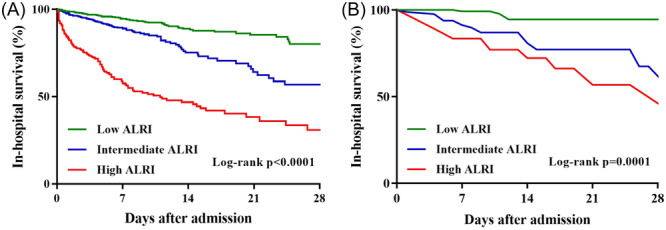

## 1 INTRODUCTION

Acute myocardial infarction (AMI) is a common emergency resulting from acute myocardial ischemia and necrosis caused by an insufficient coronary blood supply.^1^ AMI poses a serious threat to human life because of its rapidly changing conditions and high mortality rates. Despite significant advances in the diagnosis and treatment of AMI, its annual incidence in China has shown a tendency to increase. Consequently, the exploration of noninvasive markers to predict the prognosis of AMI is crucial for selecting treatment options and improving prognosis. Several novel biomarkers related to the pathophysiological processes of AMI have been identified, including inflammation, myocardial stress, neurohormonal activation, myocardial necrosis, and cardiac remodeling. Novel prognostic indicators, including soluble IL‐2,^2^ S100A12,^3^ and copeptin,^4^ exhibit relatively good predictive performance for AMI prognosis, but are not routinely detected, restricting their utility in the clinical setting.

The pathological mechanism of AMI is a form of acute ischemia and myocardial necrosis, in which necrotic myocardial cells trigger a series of inflammatory responses.^5^, ^6^ Accumulating evidence indicates that systemic inflammation aggravates atherosclerosis and the activation of peripheral leukocytes and platelets. Biomarkers for systemic inflammation are indicators of adverse clinical outcomes in patients with AMI, such as recurrent myocardial infarction and heart failure.^7^ Immune activation after AMI is conducive to cardiac remodeling,^8^ and the suppression of chronic inflammation is viewed as a treatment direction after AMI.^9^ Owing to the high mortality rate of AMI, it is important to screen all patients with AMI for hyperinflammation based on hematologic parameters and to predict early survival outcomes for better risk stratification. In this context, identifying reliable biomarkers that can reflect inflammation status is a potential predictor of AMI prognosis. The systemic immune inflammation index was reported to be a superior predictor of in‐hospital mortality compared to traditional risk factors in patients with AMI.^10^, ^11^ Karakayali et al.^12^ designed a novel systemic inflammation and nutritional status index named albumin, platelets, lymphocytes, and albumin (HALP) to predict in‐hospital mortality in patients with ST‐elevation AMI. Previous studies have shown that inflammatory markers such as C‐reactive protein and platelets can reflect the extent of the inflammatory response in patients with AMI and have been shown to be strongly correlated with the in‐hospital mortality of such patients.^13^, ^14^ An increase in aspartate aminotransferase (AST) levels on admission is regarded as an independent predictor of AMI mortality.^15^ The AST to lymphocyte ratio index (ALRI), which assesses the degree of systemic inflammation, is significantly correlated with survival outcomes in patients with hepatocellular carcinoma^16^ and metastatic colorectal cancer.^17^ Moreover, a recent study reported that the ALRI upon admission is a significant predictor of COVID‐19 mortality.^18^ However, no study has reported its predictive value for survival in patients with AMI.

In this study, we aimed to explore the correlation between ALRI and clinical features of patients with AMI and investigate the prognostic significance of ALRI using the Medical Information Mart for Intensive Care III (MIMIC‐III) database. We also assessed the predictive value of ALRI for 28‐day mortality using receiver operating characteristic (ROC) curves. Moreover, we aimed to design an AMI nomogram containing ALRI to predict 28‐day mortality in patients with AMI. Importantly, we further validated the predictive value of ALRI for 28‐day mortality using clinical data of patients with AMI from the Wuhan Sixth Hospital.

## 2 METHODS

### 2.1 MIMIC‐III database

The MIMIC‐III is a large public database comprising clinical information related to patients admitted to critical care units and was founded by the Massachusetts Institute of Technology and Harvard Medical School. The MIMIC‐III database incorporates clinical information on approximately 50,000 intensive care unit (ICU) patients, including demographic information, vital signs, multiple laboratory results, medication records, care records, and treatment data.^19^ Inclusion criteria: (1) patients with newly diagnosed AMI and first admission to ICU; (2) onset age >18 years; (3) duration of admission to the critical care unit >48 h; and (4) no clinically significant data missing. The exclusion criteria: (1) AMI individuals who died within 24 h after the onset; (2) AMI patients who received their treatment in the common ward during the whole hospitalization; and (3) AMI patients who lost follow‐up data.

We extracted the clinical and prognostic data of patients with a confirmed diagnosis of AMI, including age at diagnosis, sex, body mass index, medical history, medication history, illness severity score, laboratory findings (mean arterial pressure, leukocyte count, lymphocyte count, hemoglobin content, platelet count, albumin content, blood creatinine, total cholesterol, triglycerides, HDL‐C, LDL‐C, AST, NT‐proBNP, and troponin), length of hospitalization, and living status. The ALRI was calculated based on the ratio of AST (U/L) to lymphocytes (10^9^/L), and patients with AMI were classified into three groups: low, medium, and high ALRI, based on the 50% quartile (21.5) and 85% quartile (68.8) of ALRI. The final outcome of this clinical study was all‐cause death within 28 days of hospitalization.

### 2.2 Clinical data from the Wuhan Sixth Hospital

To further validate the prognostic role of ALRI in individuals with AMI, we retrospectively collected clinical and survival data (28‐day mortality) of patients with AMI from January 2017 to December 2020 in the Cardiology Care Unit of the Sixth Hospital of Wuhan. Moreover, all patients with AMI were admitted to the hospital for treatment within 24 h of onset, and we retrospectively collected the results of the first blood draw after the patient's admission. The clinical information of patients with AMI included demographic indices, medical history, medication history, illness severity score, and laboratory findings. The protocol for this clinical study was approved by the Ethics Committee of Sixth Hospital of Wuhan (WHSHIRB‐K‐2023007). This study was conducted in accordance with the principles of the Declaration of Helsinki. Informed consent was obtained from all subjects.

### 2.3 Construction and validation of an AMI nomogram

Significant clinical variables related to the survival outcome of AMI patients from the MIMIC‐III database were selected via univariate and multivariate Cox regression analyses. The retained optimal clinical indices with *p* values less than .05 were selected to construct the AMI nomogram. The AMI nomogram was externally validated at the Wuhan Sixth Hospital. The calibration ability of the AMI nomogram in the MIMIC‐III database and Wuhan Sixth Hospital was displayed using calibration curves. Decision curve analysis (DCA) was performed to calculate the net benefits over a range of threshold probabilities in both the MIMIC‐III database and the Wuhan Sixth Hospital.

### 2.4 Statistical methods

All statistical analyses were performed using SPSS software (version 20.0). Continuous variables that followed a normal distribution are expressed as mean ± standard deviation. For continuous variables that were not normally distributed are presented as interquartile ranges. Differences among the three groups were determined using analysis of variance. Categorical variables are expressed as frequencies with corresponding percentages, and statistical differences among the three groups were evaluated using either *χ* ^2^ or Fisher's exact tests. To determine whether ALRI was an independent risk factor for the 28‐day death in patients with AMI, Cox regression analysis was performed. ROC curves were plotted to assess the predictive power of ALRI and AMI nomograms for survival outcomes. Area under the curve (AUC) with a 95% confidence interval (CI) was selected as the quantitative index to estimate the predictive performance of the ALRI and AMI nomograms. *p* Values on both sides less than .05 were indicated as statistical difference.

## 3 RESULTS

### 3.1 Clinical relevance of ALRI in AMI individuals

A total of 2097 patients with AMI from the MIMIC‐III database were included in this study. The patients were divided into three groups according to the 50% and 85% quartiles of the ALRI. As shown in Table 1, age distribution (*p* = .006), simplified acute physiology score II (SAPSII) (*p* < .001), sequential organ failure assessment (SOFA) score (*p* < .001), blood leukocyte count (*p* = .006), platelet count (*p* = .007), length of hospitalization (*p* < .001), and in‐hospital mortality (*p* < .001) were statistically significant among the three groups based on MIMIC‐III database. Additionally, a similar analysis was conducted on 244 patients with AMI admitted to the ICU at Wuhan Sixth Hospital and divided into three groups based on their ALRI values. The results showed statistically significant differences among the three groups for blood leukocyte count (*p* = .041), platelet count (*p* = .001), and in‐hospital mortality (*p* < .001), which is vividly exhibited in Table 2.

**Table 1:** The correlation between ALRI and clinical parameters in acute myocardial infarction patients.

Clinical features	Low ALRI group	Medium ALRI group	High ALRI group	*p* Value
Demographic indexes	1045	736	316	‐
Age, years	67.2 ± 14.1	68.3 ± 14.1	70.0 ± 14.6	.006
Gender, male, *n* (%)	677 (64.8)	269 (36.5)	122 (38.6)	.530
BMI, kg/m^2^	28.0 ± 6.6	27.9 ± 6.3	27.6 ± 5.8	.157
Past medical history				
Hypertension	494 (47.3)	358 (48.6)	152 (48.1)	.847
Diabetes	278 (26.6)	200 (27.2)	92 (29.1)	.680
Heart failure	93 (8.9)	54 (7.3)	27 (8.5)	.493
Chronic kidney disease	128 (12.2)	75 (10.2)	36 (11.4)	.404
Medication history				
ACEI/ARB	489 (46.8)	346 (47.0)	147 (46.5)	.286
Aspirin	557 (53.3)	376 (51.1)	165 (52.2)	.374
Beta‐blockers	706 (67.6)	499 (67.8)	162 (51.3)	.084
Illness severity score				
APACHE II	‐	‐	‐	‐
SAPSII	34.0 ± 14.1	34.0 ± 14.9	41.9 ± 17.5	<.001
SOFA	3.7 ± 1.1	3.5 ± 1.0	5.2 ± 1.7	<.001
Laboratory findings				
MAP, mmHg	57.5 ± 12.7	57.5 ± 13.5	57.0 ± 15.2	.114
Leukocytes, ×10^9^/L	14.3 ± 6.0	13.6 ± 5.6	14.1 ± 6.4	.006
Hemoglobin, g/dL	12.7 ± 1.9	12.8 ± 1.9	12.7 ± 2.0	.286
Platelet, ×10^12^/L	262.8 ± 112.6	247.1 ± 95.5	252.3 ± 102.8	.007
Albumin, g/dL	3.4 ± 0.6	3.4 ± 0.6	3.5 ± 0.7	.667
Creatinine, mg/dL	1.4 ± 0.5	1.4 ± 0.6	1.5 ± 0.5	.096
Total cholesterol, mg/dL	161.9 ± 45.3	168.8 ± 45.7	164.4 ± 55.2	.108
Triglycerid, mg/dL	142.4 ± 75.1	140.1 ± 75.4	135.0 ± 61.3	.786
HDL‐C, mg/dL	44.6 ± 13.5	44.8 ± 12.8	44.9 ± 14.1	.433
LDL‐C, mg/dL	92.5 ± 37.7	97.1 ± 40.8	95.4 ± 41.9	.263
NT‐proBN, pg/mL	7295 (1577−19,784)	5519 (2487−16,122)	14144 (6309−20,783)	.394
TnI, ng/mL	18.8 ± 6.3	22.4 ± 6.3	16.6 ± 8.6	.103
Length of hospitalization, days	8.1 (5.1−17.3)	7.0 (4.3−14.0)	16.4 (3.6−39.6)	<.001
Hospitalization mortality, *n* (%)	75 (7.2)	95 (12.9)	142 (44.9)	<.001

Abbreviations: ACEI/ARB, angiotensin‐converting enzyme inhibitor/angiotensin receptor blocker; ALRI alanine aminotransferase to lymphocyte ratio; APACHE II, acute physiology and chronic health evaluation II; BMI, body mass index; HDL‐C, high‐density lipoprotein cholesterol; LDL‐C, low‐density lipoprotein cholesterol; MAP, mean arterial pressure; NT‐proBNP, N‐terminal pro‐brain natriuretic peptide; SAPS II, simplified acute physiology score II; SOFA, sequential organ failure assessment; TnI, troponin.

**Table 2:** The correlation between ALRI and clinical parameters in acute myocardial infarction patients from the Six Hospital of Wuhan.

Clinical features	Low ALRI group	Medium ALRI group	High ALRI group	*p* Value
Demographic indexes	126	81	37	
Age, years	64.6 ± 13.8	66.8 ± 12.0	68.6 ± 16.2	.237
Gender, male, *n* (%)	95 (75.4)	51 (63.0)	24 (64.9)	.131
BMI, kg/m^2^	24.0 ± 3.8	23.9 ± 3.4	23.6 ± 3.3	.872
Past medical history				
Hypertension	79 (62.7)	51 (63.0)	23 (62.2)	.997
Diabetes	41 (32.5)	24 (29.6)	12 (32.4)	.902
Heart failure	24 (19.0)	17 (21.0)	3 (8.1)	.069
Chronic kidney disease	18 (14.3)	13 (16.0)	7 (18.9)	.786
Medication history				
ACEI/ARB	55 (43.7)	28 (34.6)	14 (37.8)	.115
Aspirin	121 (96.0)	77 (95.1)	36 (97.3)	.848
Beta‐blockers	93 (73.8)	58 (71.6)	27 (73.0)	.723
Illness severity score				
APACHE II	7.2 ± 1.9	8.0 ± 1.8	8.7 ± 2.1	.097
Laboratory findings				
MAP, mmHg	93.2 ± 16.3	92.0 ± 17.2	90.8 ± 16.4	.722
Leukocytes, ×10^9^/L	9.5 ± 3.8	9.6 ± 3.2	11.3 ± 4.5	.041
Hemoglobin, g/dL	12.4 ± 2.1	12.1 ± 2.2	11.9 ± 2.7	.138
Platelet, ×10^12^/L	208.9 ± 62.1	190.9 ± 56.4	167.7 ± 54.9	.001
Albumin, g/dL	3.6 ± 0.8	3.6 ± 0.7	3.7 ± 0.5	.813
Creatinine, mg/dL	0.9 ± 0.3	1.1 ± 0.5	1.0 ± 0.4	.397
Total cholesterol, mg/dL	159.9 ± 35.7	156.7 ± 32.8	158.4 ± 32.8	.811
Triglycerid, mg/dL	148.1 ± 76.0	148.2 ± 74.6	142.9 ± 61.1	.793
HDL‐C, mg/dL	34.6 ± 13.0	37.7 ± 14.1	37.0 ± 12.2	.215
LDL‐C, mg/dL	102.0 ± 30.1	99.2 ± 29.0	99.6 ± 33.1	.791
NT‐proBN, pg/mL	3227 (270−11,850)	16093 (1707−33,350)	5250 (3157−12,466)	.139
TnI, ng/mL	14.7 ± 5.0	18.4 ± 6.2	18.8 ± 6.9	.064
Length of hospitalization, days	11.0 (7.0−14.0)	9.0 (6.8−13.3)	10.0 (7.5−17.8)	.463
Hospitalization mortality, *n* (%)	4 (3.2)	15 (18.5)	12 (32.4)	<.001

Abbreviations: ACEI/ARB, angiotensin‐converting enzyme inhibitor/angiotensin receptor blocker; ALRI, alanine aminotransferase to lymphocyte ratio; APACHE II, acute physiology and chronic health evaluation II; BMI, body mass index; HDL‐C, high‐density lipoprotein cholesterol; LDL‐C, low‐density lipoprotein cholesterol; MAP, mean arterial pressure; NT‐proBNP, N‐terminal pro‐brain natriuretic peptide; TnI, troponin.

### 3.2 Survival analysis

**Figure 1:**
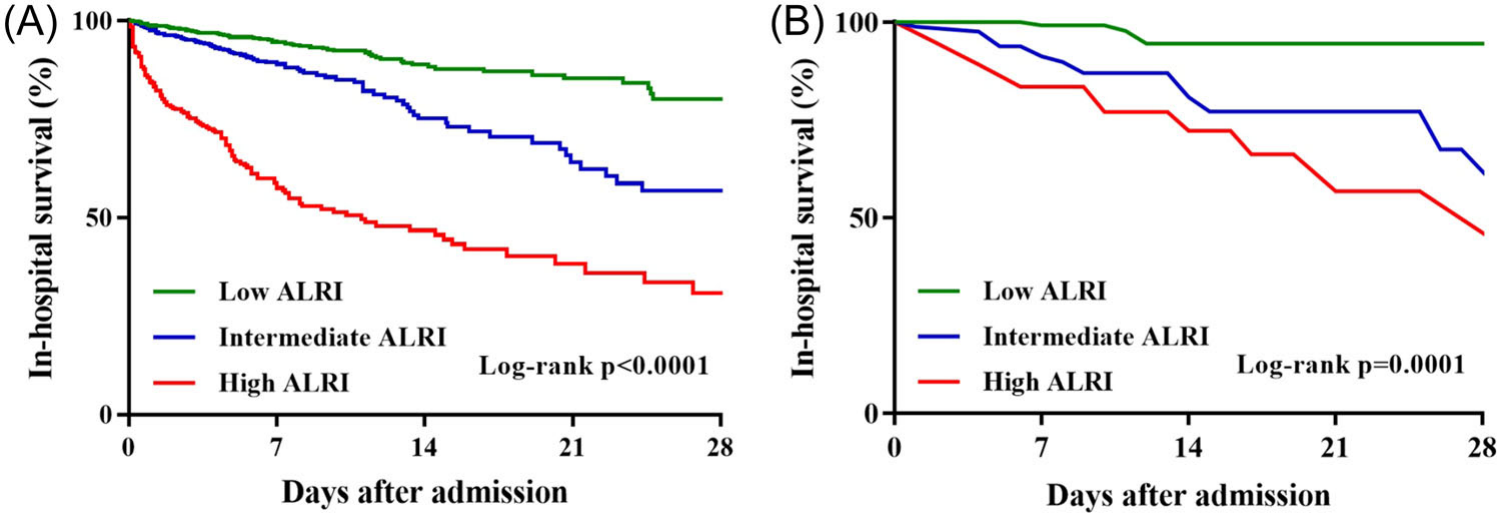
Survival analysis of 28‐day mortality in acute myocardial infarction patients stratified by low, medium, and high ALRI groups. (A) Data from MIMIC‐III database. (B) Data from the Wuhan Sixth Hospital. ALRI, aminotransferase‐to‐lymphocyte ratio index; MIMIC, medical information mark for intensive care.

As shown in Table 3, both the MIMIC‐III and Wuhan Sixth Hospital cohorts demonstrated a close correlation between in‐hospital mortality due to ALRI and AMI. Further evaluation of the correlation between the ALRI and the survival outcome of patients with AMI was conducted using Kaplan–Meier curves. Survival analysis revealed that AMI patients with a high or intermediate ALRI had significantly higher 28‐day survival rates than those with a low ALRI, as shown in Figure 1A (HR = 5.816, 95% CI: 4.293–7.879, *p* < .0001). A similar trend was observed in data from the Wuhan Sixth Hospital (HR = 8.647, 95% CI: 2.768–27.011, *p* < .001, Figure 1B), indicating that a higher 28‐day mortality was observed in AMI patients with a high ALRI than in those with a low ALRI.

**Table 3:** Cox regression analysis of factors influencing in‐hospital mortality in patients with AMI from MIMIC‐III database.

Indicators	Univariate Cox		Multivariate Cox	
	HR (95% CI)	*p* Value	HR (95% CI)	*p* Value
Age	1.031 (1.022−1.040)	<.001	1.008 (0.998−1.019)	.126
Male	0.656 (0.525−0.820)	<.001	0.982 (0.769−1.255)	.887
BMI	0.972 (0.954−1.090)	.083		
Past medical history				
Hypertension	1.011 (0.810−1.263)	.920		
Diabetes	0.818 (0.632−1.059)	.127		
Heart failure	1.139 (0.835−1.553)	.412		
Chronic kidney disease	1.263 (0.936−1.703)	.127		
Inpatient medication history				
ACEI/ARB	0.196 (0.141−0.271)	<.001	0.467 (0.324−0.672)	<.001
Aspirin	0.676 (0.541−0.846)	.001	1.247 (0.948−1.640)	.114
Beta‐blockers	0.268 (0.213−0.339)	<.001	0.407 (0.305−0.543)	<.001
Illness severity score				
SAPSII	1.197 (1.167−1.228)	<.001	1.044 (1.031−1.057)	<.001
SOFA	1.053 (1.047−1.059)	<.001	0.976 (0.925−1.029)	.359
Multiple laboratory findings				
MAP	0.962 (0.956−0.969)	<.001	0.978 (0.970−0.987)	<.001
Leukocytes	1.036 (1.025−1.046)	<.001	1.010 (0.998−1.022)	.093
Hemoglobin	0.950 (0.897−1.008)	.088		
Platelet	1.000 (0.999−1.001)	.979		
Albumin	0.942 (0.738−1.202)	.630		
Creatinine	1.174 (1.117−1.234)	<.001	0.973 (0.895−1.057)	.515
Total cholesterol	0.995 (0.989−1.000)	.064		
Triglyceride	1.000 (0.998−1.002)	.967		
HDL‐C	1.013 (0.997−1.029)	.104		
LDL‐C	0.995 (0.988−1.002)	.166		
Lg (NT‐proBNP)	1.628 (0.768−3.450)	.203		
TnI	1.005 (0.990−1.021)	.515		
ALRI				
Low	Reference	‐	Reference	‐
Medium	2.123 (1.567−2.875)	<.001	2.269 (1.646−3.127)	<.001
High	7.856 (5.933−10.401)	<.001	5.816 (4.293−7.879)	<.001

Abbreviations: ALRI, aminotransferase‐to‐lymphocyte ratio index; AMI, acute myocardial infarction; MIMIC, medical information mark for intensive care; SAPSII, simplified acute physiology score II; SOFA, sequential organ failure assessment.

### 3.3 Cox regression analysis

Cox regression analysis was used to determine whether a high ALRI was an independent risk factor for 28‐day mortality in patients with AMI. Multivariate Cox regression analysis based on the MIMIC‐III database revealed that the risk of death in the high ALRI group was 5.816‐fold higher than that in the low ALRI group (HR = 5.816, 95% CI: 4.293–7.879, *p* < .001; Table 3) after adjusting for confounding clinical factors, such as sex and age. Similarly, multivariate Cox regression analysis revealed that the mortality risk in the high ALRI group was 4.969‐fold higher than that in the low ALRI group (HR = 4.969, 95% CI: 1.529–16.152, *p* = .008) after adjusting for confounding clinical factors when validated with clinical data from the Wuhan Sixth Hospital (Table 4). Collectively, the results from the two cohorts demonstrate that elevated ALRI levels are a strong risk index for worse survival outcomes in patients with AMI.

**Table 4:** Cox regression analysis of factors influencing in‐hospital prognosis in patients with AMI from the Six Hospital of Wuhan.

Indicators	Univariate Cox regression		Multivariate Cox regression	
	HR (95% CI)	*p* Value	HR (95% CI)	*p* Value
Age	1.047 (1.017−1.078)	.002	1.022 (0.985−1.059)	.249
Male	0.505 (0.250−1.023)	.058	0.560 (0.242−1.299)	.177
BMI	0.924 (0.839−1.018)	.108		
Past medical history				
Hypertension	0.908 (0.433−1.900)	.797		
Diabetes	1.228 (0.598−2.522)	.576		
Heart failure	1.388 (0.425−4.534)	.588		
Chronic kidney disease	2.716 (1.300−5.675)	.008	1.782 (0.781‐4.072)	0.170
Inpatient medication history				
ACEI/ARB	0.412 (0.184−0.922)	.031	0.509 (0.213−1.224)	.127
Aspirin	0.292 (0.088−0.969)	.044	0.401 (0.100−1.609)	.197
Beta‐blockers	0.285 (0.133−0.609)	.001	0.408 (0.177−0.940)	.036
Illness severity score				
APACHE II	1.156 (1.079−1.238)	<.001	1.126 (1.007−1.261)	.040
Multiple laboratory findings				
MAP	0.996 (0.975−1.108)	.721		
Leukocytes	0.961 (0.869−1.063)	.437		
Hemoglobin	0.800 (0.696−0.919)	.002	1.089 (0.905−1.311)	.366
Platelet	1.000 (0.994−1.005)	.862		
Albumin	1.169 (0.723−1.889)	.524		
Creatinine	1.393 (1.188−1.634)	<.001	1.204 (0.947−1.531)	.129
Total cholesterol	1.003 (0.993−1.013)	.599		
Triglyceride	0.999 (0.994−1.004)	.689		
HDL‐C	1.016 (0.991−1.042)	.213		
LDL‐C	1.001 (0.989−1.013)	.853		
Lg (NT‐proBNP)	1.397 (0.757−2.576)	.284		
TnI	0.999 (0.969−1.030)	.951		
ALRI				
Low	Reference	‐	Reference	‐
Medium	5.232 (1.731−15.813)	.003	3.299 (1.043−10.439)	.042
High	8.647 (2.768−27.011)	<.001	4.969 (1.529−16.152)	.008

Abbreviations: ALRI, aminotransferase‐to‐lymphocyte ratio index; AMI, acute myocardial infarction.

### 3.4 Predictive power of ALRI

**Figure 2:**
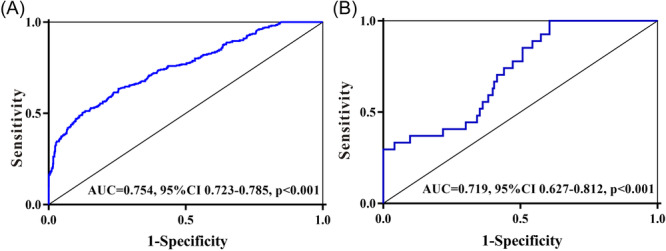
Predictive value of ALRI for the 28‐day mortality of patients with acute myocardial infarction. (A) Data from MIMIC‐III database. (B) Data from the Sixth Hospital of Wuhan. ALRI, aminotransferase‐to‐lymphocyte ratio index; AUC, area under the curve; MIMIC, medical information mark for intensive care.

Given that ALRI is an independent risk factor for in‐hospital survival, we constructed ROC curves to assess the predictive power of ALRI for the 28‐day mortality in patients with AMI. Our results showed that the predictive power of ALRI presented an AUC of 0.754 (95% CI: 0.723–0.785) in the MIMIC‐III database (Figure 2A), with a sensitivity of 87.6% and specificity of 60.7%. When validated with data from the Sixth Hospital of Wuhan (Figure 2B), the predictive power of ALRI presented an AUC of 0.719 (95% CI: 0.627–0.812), with a sensitivity‐and specificity of 78.9% and 61.6%, respectively. In summary, we concluded that a high ALRI exhibited good predictive power for a higher death risk in individuals with AMI.

### 3.5 Predictive performance of AMI nomogram

**Figure 3:**
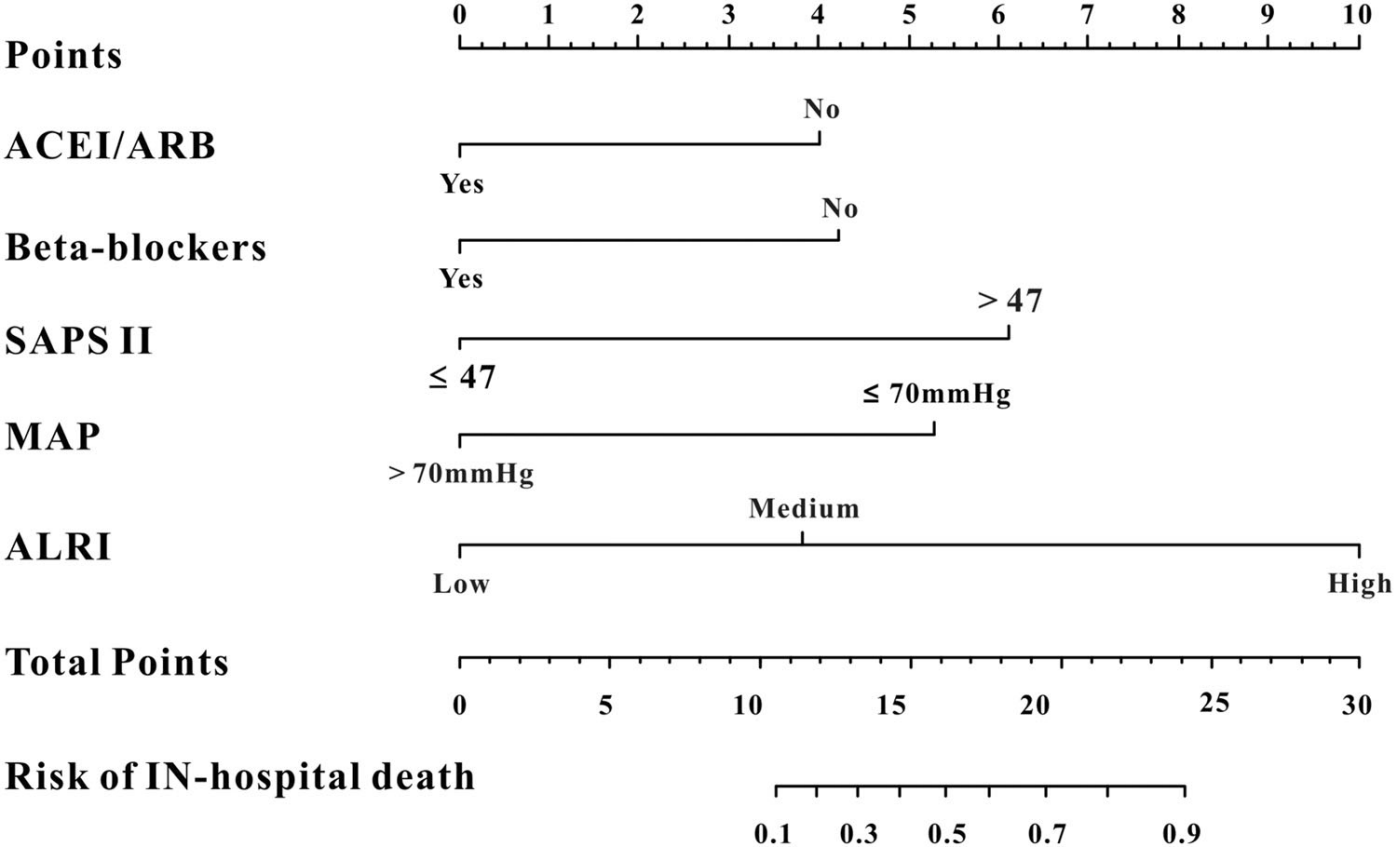
The acute myocardial infarction (AMI) nomogram for predicting the 28‐day mortality in AMI individuals. ACEI/ARB, angiotensin‐converting enzyme inhibitor/angiotensin receptor blocker; ALRI, aminotransferase‐to‐lymphocyte ratio index; MAP, mean arterial pressure; SAPSII, simplified acute physiology score II.

**Figure 4:**
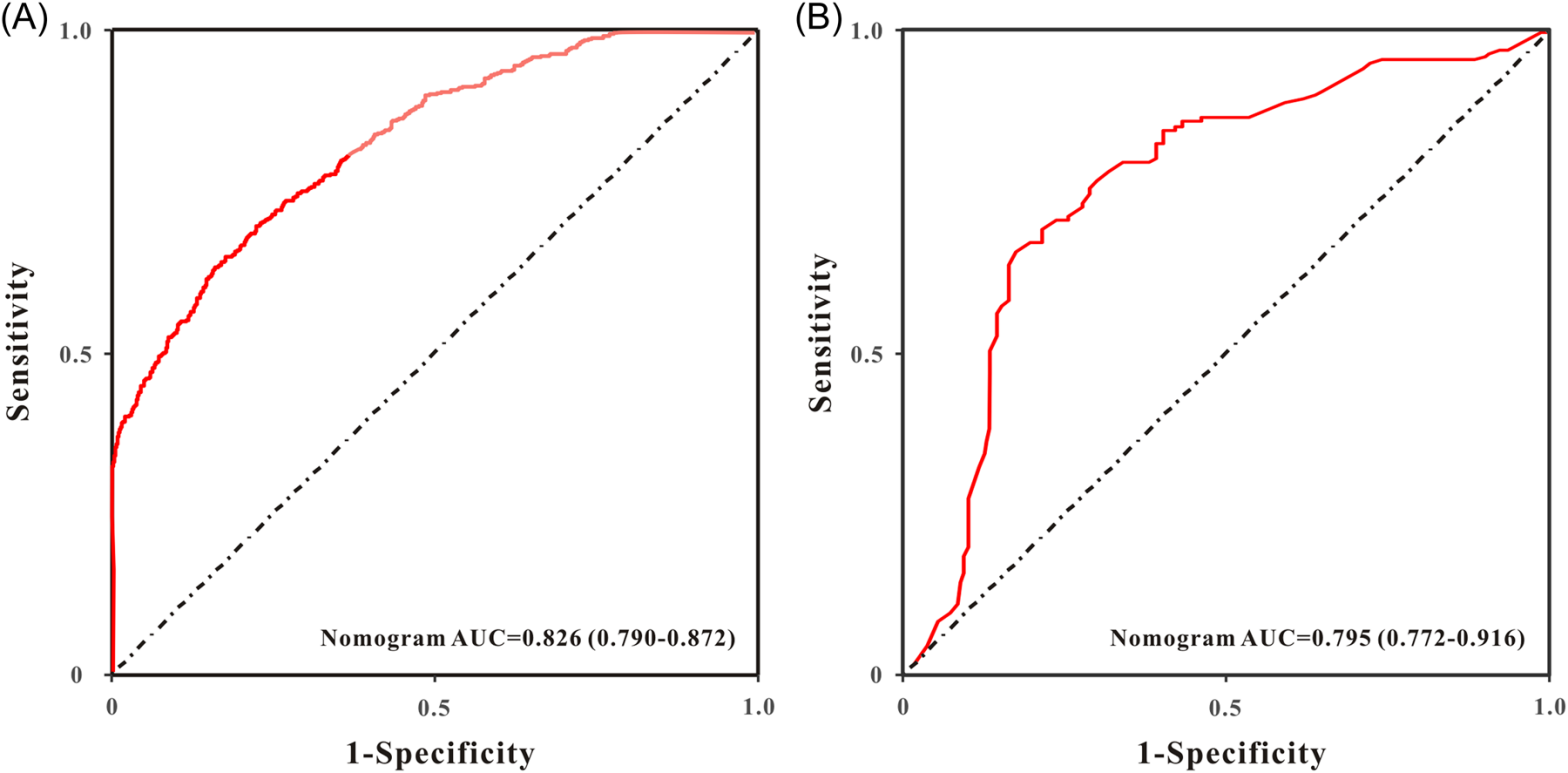
The receiver operating characteristic (ROC) plots of the acute myocardial infarction nomogram for predicting the 28‐day mortality in AMI individuals. (A) MIMIC‐III database. (B) Wuhan Sixth Hospital. AMI, acute myocardial infarction; AUC, area under the curve; MIMIC, medical information mark for intensive care.

**Figure 5:**
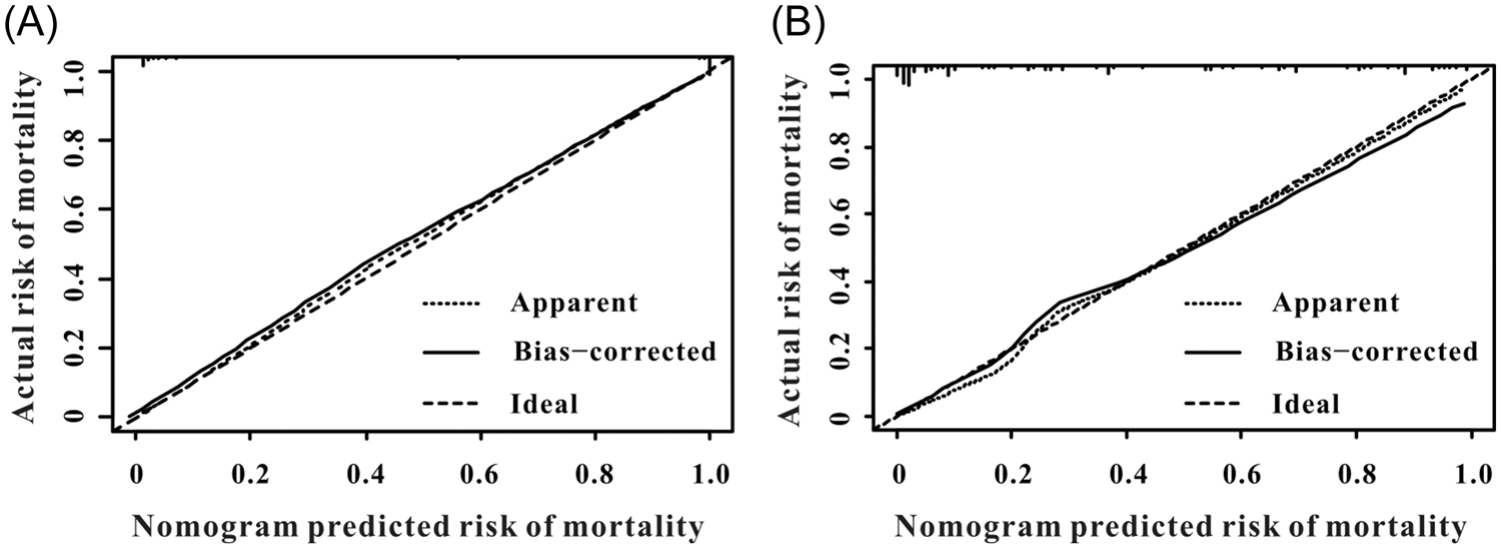
Calibration curves for predicting the 28‐day mortality in AMI individuals. (A) The MIMIC‐III database. (B) Wuhan Sixth Hospital. AMI, acute myocardial infarction; MIMIC, medical information mark for intensive care.

**Figure 6:**
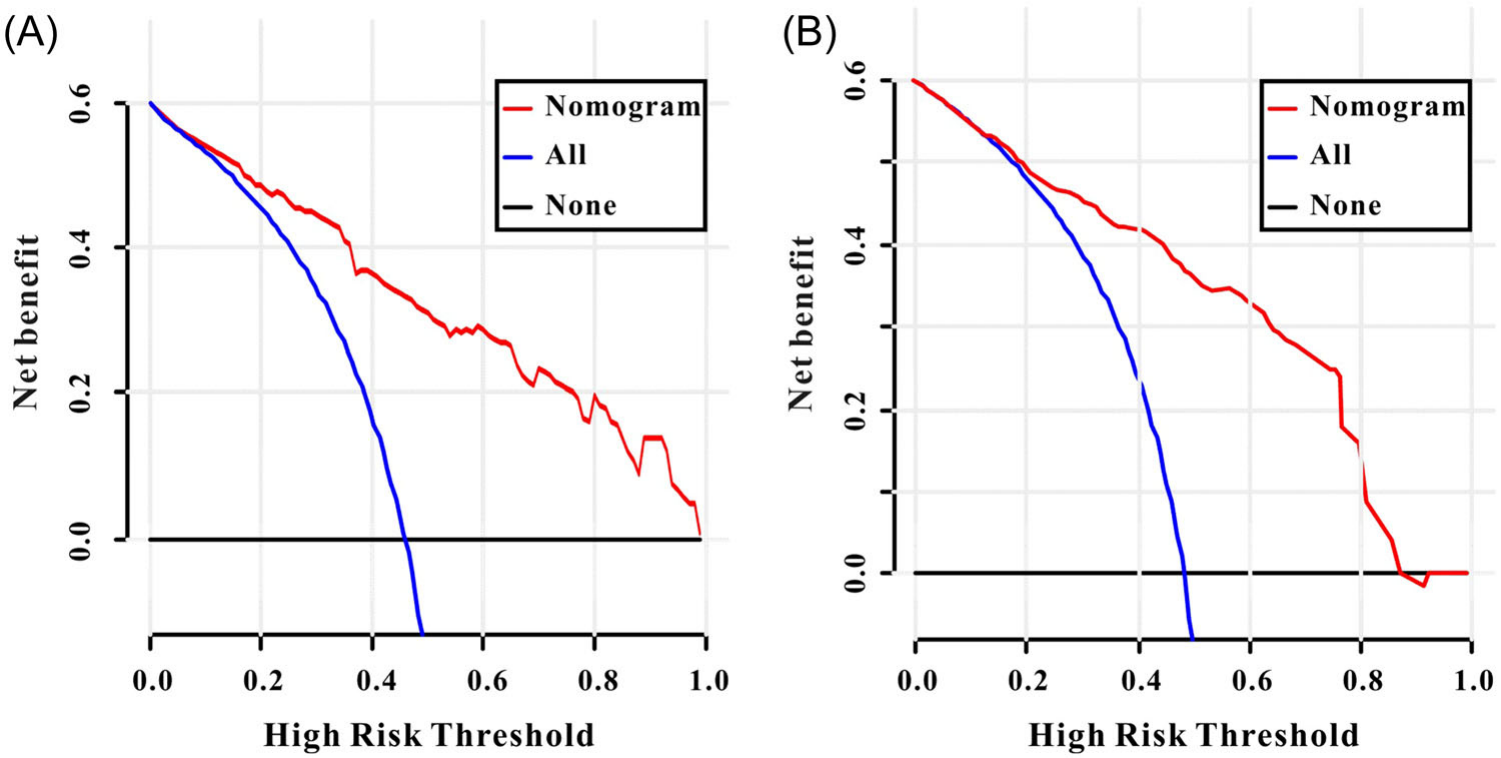
The decision curves analysis (DCA) curves of the AMI nomogram. (A) MIMIC‐III database. (B) Wuhan Sixth Hospital. AMI, acute myocardial infarction; MIMIC, medical information mark for intensive care.

In the MIMIC‐III database, the significant risk factors after multivariate Cox regression analysis were the use of ACEIs/ARBs, beta‐blockers, SAPS‐II, MAP, and ALRI. An AMI nomogram was created to display the variables and their corresponding points (Figure 3). ROC curves were used to measure the predictive ability of the AMI nomogram for survival. As shown in Figure 4A, the AUC of 28‐day mortality reached 0.826 (95% CI: 0.79–0.872) in the MIMIC‐III database. When validated with clinical information from the Wuhan Sixth Hospital (Figure 4B), the AUC of the 28‐day mortality was 0.795 (95% CI: 0.772–0.916). The calibration curves of the AMI nomogram displayed good consistency between the actual 28‐day mortality and predicted 28‐day mortality not only in the MIMIC‐III database (Figure 5A) but also in the Wuhan Sixth Hospital (Figure 5B). Finally, DCA curves (Figure 6A,B) demonstrated that the AMI nomogram obtained a positive net benefit from the risk of 28‐day death, indicating the great clinical value of the AMI nomogram in predicting the 28‐day mortality of AMI patients.

## 4 DISCUSSION

AMI remains the leading cause of death due to cardiovascular diseases in China. Investigating new biomarkers closely associated with survival outcomes is important for the early risk assessment of patients with AMI. In this study, we investigated the correlation between ALRI and clinical parameters in patients with AMI based on the MIMIC‐III database and evaluated the predictive value of ALRI for 28‐day mortality in patients with AMI. Our findings showed that AMI patients with a high ALRI had a significantly higher 28‐day mortality than those with a lower ALRI. Moreover, we validated the close correlation between ALRI and 28‐day mortality by analyzing data from Wuhan Sixth Hospital. Our results demonstrate that the ALRI has a high predictive value for 28‐day mortality in patients with AMI and can serve as a noninvasive prognostic indicator for the risk stratification of patients with AMI.

A meta‐analysis^20^ revealed that high uric acid level was correlated with an increased in‐hospital mortality risk of AMI patients (RR: 2.10, 95% CI: 1.03–4.26). Another study reported that a long prothrombin time was a significant risk factor for all‐cause mortality in AMI patients (HR: 4.04; 95% CI: 2.83–5.75).^21^ A recent clinical study found that hyperstimulating hormone elevation was a significant predictor of all‐cause mortality in individuals with AMI (HR: 1.560; 95% CI: 1.017–2.392).^22^ Our study assessed the prognostic role of ALRI derived from blood routine and liver function, and found that high level of ALRI was an important risk factor for 28‐day survival in patients with AMI (HR = 5.816, 95% CI: 4.293−7.879), indicating that ALRI is an reliable and potent prognostic factor for AMI individuals. In addition, we also validated the prognostic association with an independent cohort and also obtained the nice predictive performance.

With the establishment of chest pain centers in hospitals, significant advances have been made in AMI treatment. Emergency physicians and cardiologists face substantial decision‐making risks. Inaccurate assessment of the prognosis of patients with AMI impedes the selection of appropriate treatment strategies. Previous studies explored the correlation between interleukin‐8,^23^ NT‐proBNP,^24^ TRPV6,^25^ and AMI prognosis. However, a small sample size limits the predictive value of these indices. Using the MIMIC‐III database, one of the largest critical care databases worldwide, we explored the clinical correlation between ALRI and clinical parameters in patients with AMI. MIMIC‐III provides a high‐quality large‐sample data platform that enables the application of classical statistical methods. The freely accessible nature of the MIMIC‐III database improves the comparability of research findings. However, while it contains clinical data solely from American patients, Therefore, we validated our results with real‐world data from Chinese patients at the Wuhan Sixth Hospital and explored the predictive value of ALRI for AMI prognosis in multiple populations.

AMI can lead to injury or even death of myocardial cells, followed by a strong inflammatory reaction in myocardial tissues. An excessive inflammatory reaction aggravates the apoptosis of myocardial cells and triggers serious adverse events, such as arrhythmia or even sudden death.^26^ The systemic immune‐inflammation index has been proven to be a reliable predictor of worse outcomes in patients with AMI, and its predictive power is significantly greater than that of the platelet‐to‐lymphocyte ratio.^27^, ^28^ Karakayali et al.^29^ reported that the white blood cell count to mean platelet volume ratio correlated with the syntax score in ST elevation AMI. Moreover, Ji et al.^30^ found that the neutrophil‐to‐lymphocyte ratio is a reliable indicator of in‐hospital death in patients with AMI. It is necessary to search for new types of inflammatory biomarkers that can accurately predict in‐hospital death in patients with AMI.^31^ ALRI is also a combined inflammatory index, while we have known nothing about its role in AMI.

AST originates from the myocardium and other tissues such as the liver. Under normal conditions, serum AST levels remain low. The AST/alanine aminotransferase (ALT) ratio was reported to correlate with the coronary slow‐flow phenomenon.^32^ However, during AMI or acute myocarditis, cellular membrane permeability increases due to damaged myocardial cells, releasing intracellular AST into the bloodstream and elevating the serum AST concentration.^33^ Importantly, increased AST levels correlated strongly with the degree of myocardial injury and extent of infarction. Elevated serum AST levels may reflect the severity of AMI. Recent research has demonstrated that as AMI triggers the immune response, lymphocyte infiltration increases significantly.^34^ Studies have revealed that CD4+ regulatory T cells, especially CD4+ T cells, may benefit wound healing following AMI, whereas depleted B lymphocytes interfere with repair.^35^ After myocardial infarction, autoantigen recognition is necessary for T cell activation, which is vital for the immune response to myocardial injury.^36^ Moreover, The main stream opinion is that lymphocyte plays the protective role in AMI. Xia et al.^37^ reported that regulatory T cells in the heart potentiate cardiac protection from myocardial infarction. Wu et al.^38^ deemed that a distinct “repair” role of regulatory T cells in AMI. Hence, a decreased lymphocyte count will induce a higher ALRI value, which indicates less favorable survival outcomes in patients with AMI. Consequently, ALRI is typically considered an inflammatory marker^39^ that is convenient to obtain and inexpensive in a clinical setting. Moreover, ALRI is not influenced by dehydration and possesses a high predictive value for survival outcomes in individuals with AMI.

Although this is the first clinical report related to ALRI in individuals, there were still three limitations to our analysis. First, this was a retrospective trial, and bias was inevitable. Thus, the sample size of AMI patients from Wuhan Sixth Hospital was small, and more AMI patients from multiple medical centers should be included in the future. Finally, 28‐day mortality is a short‐run survival outcome, and the long‐term effects of ALRI on patients from the long term, such as 90‐ and 180‐day mortality, were not investigated owing to its retrospective nature.

## 5 CONCLUSION

Based on routine examination indicators for patients with AMI such as AST and lymphocyte count, ARLI is a cost‐effective and easily obtainable inflammatory marker. Our study utilized a large sample of AMI patients to demonstrate that ALRI is related to multiple clinical indicators in AMI patients, and that individuals with high ARLI levels have worse survival outcomes. Our findings indicate that ALRI may be a reliable and easily accessible biomarker for early risk stratification in patients with AMI.

## AUTHOR CONTRIBUTIONS

**Huidi Liu**: Conceptualization; methodology; and writing. **Fan Zhao**: Data collection; validation; and editing. **Jun Yin**: Methodology; and software and formal analyses. **Taimin Liu**: Investigation and revision. **Bo Liu**: Supervision; reviewing; and editing.

## CONFLICT OF INTEREST STATEMENT

The authors declare no conflict of interest.

## ETHICS STATEMENT

The protocol for the clinical study was approved by the ethics committees of the Sixth Hospital of Wuhan (WHSHIRB‐K‐2023007). The study was conducted in accordance with the Declaration of Helsinki. The informed consent was obtained from all subjects.

## Data Availability

The original data will be available on reasonable request from the corresponding author.
